# Meeting of Dentists—Resolutions of Respect

**Published:** 1869-02

**Authors:** 


### MEETING OF DENTISTS—RESOLUTIONS OF RESPECT.

At an adjourned meeting, held at Dr. Wright's office, March 1, 1869, by the members of the Dental profession of the City of Columbus, called for the purpose of considering and passing some befitting resolutions expressive of our bereavement in the death of a professional brother, Dr. John Fowler, whose deportment in life as a citizen and professional brother has endeared him to all our hearts, on motion of Mr. Dunn, the following resolutions were unanimously adopted:

WHEREAS, In the infinite wisdom of Almighty God He has seen fit to take from us our beloved friend and brother, Dr. John Fowler.

WHEREAS, By his honesty of purpose, by his integrity of character and by the exercise of the most exalted, social, moral and professional qualities, he has left an example most worthy of our imitation; it is, therefore, by this meeting,

*Resolved*, That in this dispensation the Dental profession has sustained a loss which will be felt and deeply mourned by each and all its members.

*Resolved*, That we cordially tender to the family of the deceased our deepest sympathy and condolence, and our prayers that they may be sustained and comforted by Him who, for his own wise purpose, has called our brother hence.

*Resolved*, That a copy of these resolutions be sent for publication in the different Dental journals, and to each of the daily papers of our city, and that a copy of the same be transmitted to the family of our deceased brother.

H. TODD, W. W. RILEY,
E. M. WRIGHT, J. B. BEAUMAN,
G. W. DUNN, R. G. WARNER,
ALLEN F. EMMINGER, A. SPENCER.
D. MCBRIAR,

